# Evaluation of Volatilomic Fingerprint from Apple Fruits to Ciders: A Useful Tool to Find Putative Biomarkers for Each Apple Variety

**DOI:** 10.3390/foods9121830

**Published:** 2020-12-09

**Authors:** Sonia Medina, Rosa Perestrelo, Regina Pereira, José S. Câmara

**Affiliations:** 1 CQM–Centro de Química da Madeira, Campus da Penteada, Universidade da Madeira, 9020-105 Funchal, Portugal; rmp@staff.uma.pt (R.P.); jsc@staff.uma.pt (J.S.C.); 2 Department of Food Science and Technology, Research Group on Quality, Safety and Bioactivity of Plant Foods, CEBAS (CSIC), Campus Espinardo, 30100 Murcia, Spain; 3 Direção Regional de Agricultura, Divisão de Inovação Agroalimentar, Avenida Arriaga, n° 21A, Edificio Golden Gate, 9000-060 Funchal, Portugal; regina.pereira@madeira.gov.pt; 4 Departamento de Química, Faculdade de Ciências Exatas e Engenharia, Campus da Penteada, Universidade da Madeira, 9020-105 Funchal, Portugal

**Keywords:** apple fruits, juices, ciders, volatile signature, HS–SPME/GC–MS, chemometric tools

## Abstract

Aroma is a crucial criterion to assess the quality of apple fruits, juices, and ciders. The aim of this study was to explore similarities and differences in volatile profiles among apple fruits, juices, and ciders from different apple varieties (Festa, Branco, and Domingos) by headspace solid-phase microextraction gas chromatography–mass spectroscopy (HS–SPME/GC–MS). A total of 142 volatile organic compounds (VOCs) were identified, but only 9 were common in all analysed matrices and apple-tested varieties. Esters, alcohols, and aldehydes presented a higher concentration in apple fruits and juices, whereas esters, alcohols, and acids were dominant in ciders. Moreover, there were unique VOCs for each matrix and for each variety, highlighting the importance of the selection of apple varieties as an important factor to obtain good sensory and quality ciders, multiple benefits, and legal protection against the misuse of local products.

## 1. Introduction

Apple aroma is a crucial criterion to assess fruit quality. This organoleptic quality is due to several volatile organic compounds (VOCs) such as esters, alcohols, aldehydes, ketones, acids, and esters [1]. More specifically, 2-methyl butyl acetate, butyl acetate, and (***E***)-2-hexenal are reported as the most significant VOCs contributing to the typical apple aroma [2]. Most VOCs in apple juice are not genuine constituents of apples, but produced from precursors by enzymatic reactions upon squeezing [3,4]. Apple variety, environment, ripening stage, storage, and processing procedure are some factors that influence the content of VOCs in apple juices [3]. Consequently, cider, a globally popular beverage, is obtained from the partial or total alcoholic fermentation of apple juice (raw material) [5]. The level of VOCs in ciders depends on the applied technology, the microorganisms involved in the fermentation process, ageing on lees, maturation, and storage conditions [4,6,7]. Typically, ripe or overripe (senescent) apples are used in cider processing due to their softened structures, thus resulting in higher juice yields and an increase in sugar content during apple ripening [8]. In fact, in a recent study, for all studied varieties, senescent fruits provided more aromatic fermented apple beverages [9]. The VOCs formed during the fermentation process, such as 3-methyl-1-butanol, 2-phenylethanol, ethyl butanoate, ethyl hexanoate, ethyl octanoate, ethyl decanoate, ethyl 2-methylbutanoate, 3-methylbutyl ethanoate, hexanoic acid, octanoic acid, and 2-methyl butanoic acid, are responsible for the fruity odours of ciders [10,11,12].

Currently, the food-quality programme of the European Union encourages food-origin protection through Protected Designation of Origin (PDO) and Protected Geographical Indication (PGI)) with the purpose of ensuring the quality of the final product [7]. Therefore, an analytical approach combined with a microextraction procedure should be developed to guarantee the genuineness of foodstuffs. An analytical tool widely used in the establishment of the volatile profile of apples, juices, and ciders is gas chromatography–mass spectrometry (GC–MS) combined with headspace solid-phase microextraction (HS–SPME) in order to define the authenticity and typicity of the samples using VOCs [4,11,13,14]. This method is a valuable tool in the establishment of a volatile profile since it requires small amounts of a sample and no solvents, and it is fast, economical, reproducible, and sensitive. 

Concerning the evaluation of the authenticity and typicity of apple-related products using VOCs, Medina et al. [13] used HS–SPME/GC–MS combined with chemometric tools (e.g., principal-component analysis (PCA)) to characterize the volatile fingerprint of apple juices from the island of Madeira. The obtained results revealed that VOCs could be used as authenticity markers to validate the variety and geographical origin of apple juices, providing local producers with numerous benefits. Perestrelo et al. [11] also established the volatile signature of apple ciders from five different geographical regions of Madeira by HS–SPME/GC–MS combined with chemometric tools. In the same context, Nespor et al. [14] evaluated the technology used in cider making, and differences in VOC composition were observed between ciders produced under intensified and traditional technologies. Despite the high potentiality of this methodology, few studies were performed to explore the similarities and differences of volatile profiles among apple fruits, juices, and cider samples. In this sense, the aim of this study was to establish the volatile profile of these apple matrices (fruits, juices, and ciders), from different apple varieties (Festa, Branco, and Domingos) from Madeira using HS–SPME/GC–MS. Then, the obtained data were subjected to the chemometric approach in order to find putative markers of apple variety.

## 2. Materials and Methods 

### 2.1. Chemicals and Materials

All reagents utilized in this assay were of analytical quality. Sodium chloride (NaCl, 99.5%), and calcium chloride (CaCl_2_, >99.0%) were obtained from Panreac (Spain, Barcelona). Ultrapure water was supplied from a Milli-Q^®^ system (Millipore); the 3-octanol used as internal standard and other VOCs for identification, namely, 1-butanol, 1-heptanol, 1-octanol, 1-propanol, (E)-2-hexenal, 2-methyl-butanal, 2-ethyl-1-hexanol, 2-methyl-1-propanol, 2-phenylethanol, acetaldehyde, benzaldehyde, ethanol, ethyl acetate, ethyl butanoate, ethyl decanoate, ethyl hexanoate, ethyl octanoate, ethyl propanoate, ethyl 3-hydroxybutanoate, hexyl acetate, and pentanal with purity up to 98% were acquired from Sigma Aldrich (Madrid, Spain). Helium gas purity of 5.0 (Air Liquide, Portugal) was utilized as the GC carrier gas. Solid-phase microextraction holder and divinylbenzene/carboxen/polydimethylsiloxane (DVB/CAR/PDMS) fiber were supplied from Supelco (Bellefonte, PA, USA). The Kovats index (KI) was calculated by the injection of a series of C_8_ to C_20_ straight-chain n-alkanes (at 40 mg L^−1^ in n-hexane) produced by Fluka (Buchs, Switzerland).

### 2.2. Apple-Fruit Samples

Fresh Festa, Branco, and Domingos apple varieties (*Malus domestica*) were directly provided by local producers from Jardim da Serra (JS), which is a Madeira region located within coordinates 32°41′49.00″ N and −16°59′39.00″ W, with an average temperature of 14.7 °C, mean annual rainfall of 690 mm, and altitude of 825 m. The chosen varieties for this study are the most cultivated varieties in the JS region. All samples from the same variety, produced in 2017, were collected (~3 kg of each variety) in the senescent ripening stage from 5 different trees in order to achieve as representative a sample as possible, and they were visually inspected to ensure no apparent damage or disease. The maturation index was determined (no more than 24 h after collection) using the starch–iodine test according to Blanpied et al. [15], using a reference colour–number chart, 8 being the number for senescent (over-ripe) apples.

### 2.3. Sample Processing

#### 2.3.1. Apple-Fruit Processing

The apples of each variety were cleaned with tap water, unpeeled, and deseeded. With the purpose of homogenizing the apple-fruit samples, all apple pieces were immediately transferred into a blender. An amount of CaCl_2_ (3%, *w*/*v*) was added to avoid enzyme browning according to a previous study [16]. The mixture was stored in glass vials at 4 °C until analysis. 

#### 2.3.2. Apple-Juice Processing 

Other servings of apple fruits (cleaned, unpeeled, deseeded, and cut into pieces) were squeezed at room temperature (22 ± 1 °C) using a hand press juicer machine for apples. An amount of CaCl_2_ (3%, *w/v*) was added. In order to obtain a representative portion of the apple juice of a specific variety, several apple fruits of the same variety were used to make a whole juice. In this study, fresh (nonthermal processing) apple juices were used to collect data on the aroma descriptors of these juices that could be used as useful information in the traditional cider-making process. The obtained juice was divided into aliquots of 50 mL and stored in sealed glass bottles at 4 °C until further analysis. 

#### 2.3.3. Cider Processing 

The cider (fermented apple beverage) samples were produced in 2017 for each variety (not blending) according to traditional fermentation methods in open stainless-steel tanks (100 L) at 14 ± 1 °C over the course of 3 weeks and in direct contact with lees. These ciders (from Festa, Branco, and Domingos apple varieties) were provided by specific producers (3 bottles of 750 mL for each variety) from JS, and they were obtained through fermentation by commercial *Saccharomyces cerevisiae* Bouquet yeast strains supplied by ENARTIS Portugal, LDA (Porto, Portugal), in an active dried form that was rehydrated and inoculated (20 g 100 L^−1^). After fermentation, sulphites (SO_2_) with antimicrobial and antioxidant activities were added at 30 mg L^−1^, and cider maturation (no more than 3 months) took place in stainless-steel tanks at a temperature of 14 ± 1 °C. Then, ciders were bottled in a dark glass after clarification that naturally occurred as the ciders stetted during maturation. The final product was transported to the laboratory in a cooler with ice and kept at 4 °C until chemical analysis for a maximum of 1 month.

### 2.4. Headspace Solid-Phase Microextraction

The headspace solid-phase microextraction (HS–SPME) procedure was adopted from a previous study validated in our laboratory with apple fruits [1], with slight modifications. In short, 5 g of apple fruit or 5 mL of apple juice or cider, 5 µL of 3-octanol (as IS to the concentration of 2.94 µg L^−1^), 2 g of NaCl were added into an amber glass with constant magnetic stirring of 500 rpm. Before using, the SPME fiber was conditioned according to the manufacturer’s instructions and exposed to the headspace for 45 min at 40 ± 1 °C. Then, the fiber was removed from the glass vial and immediately inserted into the GC injector port for 6 min at 250 °C for the thermal desorption of the VOCs. All analyses were performed in triplicate (*n* = 3).

### 2.5. Gas Chromatography–Quadrupole Mass Spectrometry Conditions

Chromatographic separation conditions were adopted from previous reports carried out in different apple matrices by our research team [13] using an Agilent 6890N (Palo Alto, CA, USA) gas chromatography system equipped with a BP-20 (30 m × 0.25 mm i.d. × 0.25 µm film thickness) fused silica capillary column acquired from SGE (Darmstadt, Germany) with helium (Helium N60, Air Liquid, Portugal) as carrier gas at 1 mL min^−1^ (column-head pressure: 13 psi). The temperature of the injector was set at 250 °C, and a splitless injector equipped with an insert of 0.75 mm i.d. was used. The temperature programme was fixed as follows: initial temperature of 40 °C, a ramp of 3 °C min^−1^ to 220 °C, and constant temperature was kept for 10 min at the end. The GC–qMS interface was held at 220 °C, and the manifold and quadrupole temperatures were both set at 180 °C. For MS detection, an Agilent 5975 quadrupole inert mass selective detector was used with an electron-impact (EI) energy of 70 eV and source temperature of 180 °C. The electron multiplier was set up to the autotune procedure, and acquisition mass range was set from *m/z* 30 to 300. The identification of VOCs was performed by comparing GC retention time and mass spectra with those of the standard, when available (Table 1); all mass spectra were also compared with the data library (NIST, 2005 software, Mass Spectral Search Program v.2.0d; Washington, DC, USA). The match factor criterion for identification was higher than 80%; Kovats index (KI) values were calculated according to the Van den Dool and Kratz equation [17]. Values were contrasted with values reported in the scientific literature for similar columns (Bianchi, 2007; Ferreira 2009), and with databases available online (The Pherobase and Flavornet). Semiquantification was performed, and VOC concentration was estimated in comparison to the added amount of 3-octanol (used as IS) according to the following equation: VOC concentration = (VOC GC peak area/IS GC peak area) × IS concentration. This approach was performed in a previous scientific study of Madeira wines [18]. Analyses were performed in triplicate, and average values of concentration (µg kg^−1^ (fruits) and µg L^−1^ (juices and ciders)) were used in further data analysis. Total ion chromatograms obtained by HS-SPME/GC–qMS analysis of apple fruits, juices, and ciders of the different varieties are shown in Figure S1.

### 2.6. Statistical-Data Elaboration

All experiments were carried out in triplicate, and the relative concentration is presented as mean ± standard deviation (SD). Statistical analysis was completed by use of SPSS software version 25.0 (SPSS Inc., Chicago, IL, USA) by which one-way analysis of variance (ANOVA) and the multiple-range (Tukey’s) test were performed to identify significant differences among the three matrices (fruits, juices, and ciders) and among the three varieties (Festa, Branco and Domingos). Significant differences were set at *p* < 0.05. Before applying the chemometric approach, data from GC–qMS analyses were median-normalized and Pareto-scaled [19]. Principal-component analysis (PCA) was used for unsupervised analysis, and partial least-squares discriminant analysis (PLS-DA) for supervised analysis. All features with a variable-importance-in-projection (VIP) score higher than 1.6 and differentially expressed in univariate analysis were considered to be potential biomarkers for the discrimination of samples on the basis of apple matrices (fruits, juices, and ciders). Hierarchical-clustering analysis (HCA) was generated by Ward and Euclidean distance in order to identify clustering patterns. Statistical analysis was performed using web-based application MetaboAnalyst v. 4.0, created at the University of Alberta, Canada [20].

## 3. Results

### 3.1. Qualitative and Semiquantitative Volatile Profile

**Figure 1:**
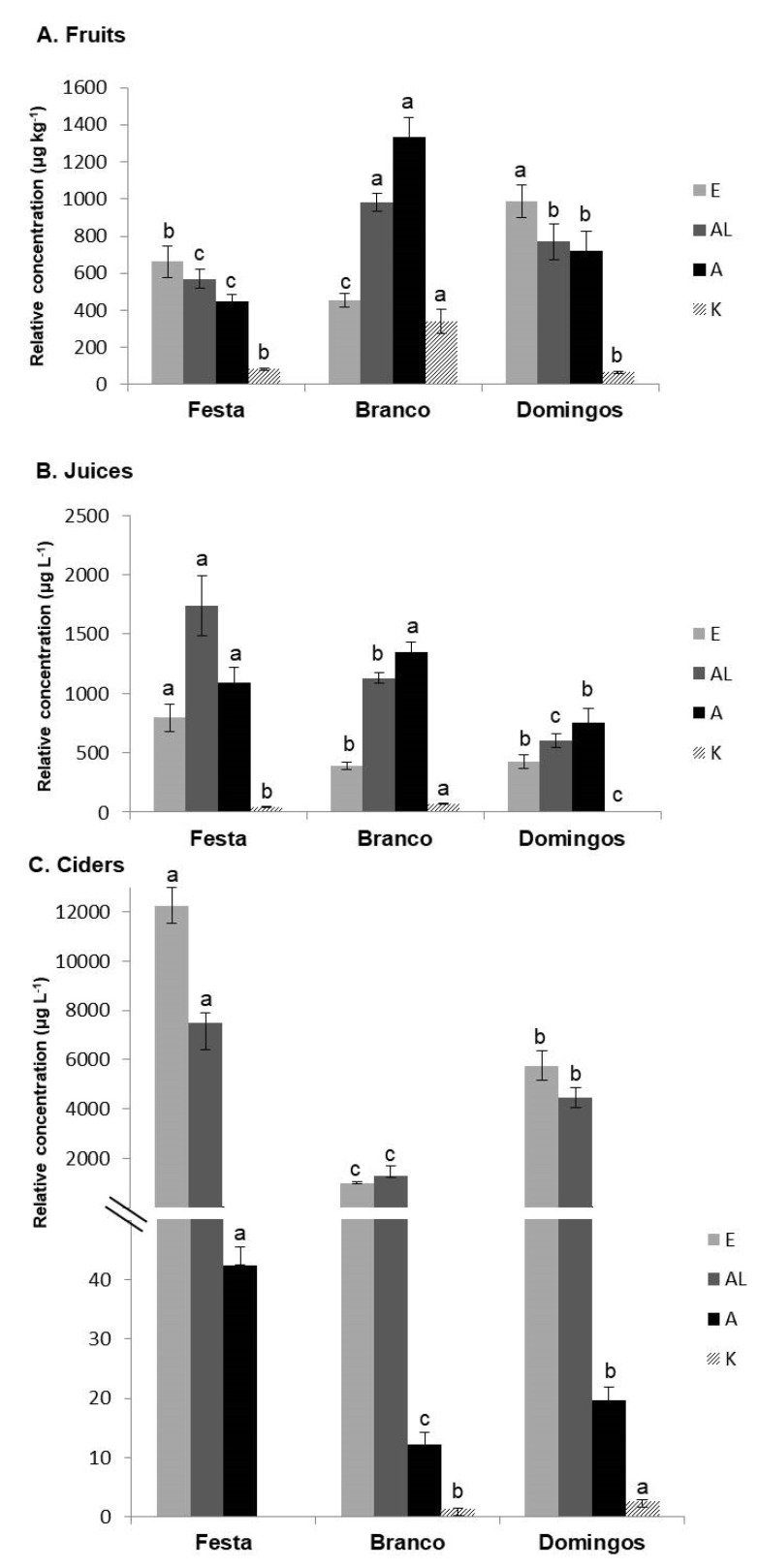
Bar graphics comparing apple (**A**) fruit, (**B**) juice, and (**C**) cider samples. Different lowercase letters indicate significant differences among varieties for the same chemical family (E, esters; AL, alcohols; A, aldehydes; K, ketones) at *p* < 0.05 according to analysis of variance (ANOVA) and multiple-range Tukey test.

In the current study, the volatile composition detected in apple-fruit, -juice, and -cider samples (142 VOCs) was characterized by the presence of 58 esters, 34 alcohols, 19 aldehydes, 10 ketones, 8 terpenoids, 7 acids, 3 sulphur compounds, 1 dioxolane, 1 lactone, and 1 aromatic hydrocarbon (Table 1). In apple fruits and juices, major chemical families were esters (on average, 24.45% and 18.82% for the total volatile composition, respectively), alcohols (24.36% and 37.94%), and aldehydes (24.60% and 37.23%). Nevertheless, an exception was observed for Branco apple fruits, since the contribution of terpenes (18.71%) for the total volatile profile was higher than that of esters (9.92%). On the other hand, esters (51.12%), alcohols (42.92%), and acids (5.29%) were the predominant chemical families in ciders (Figure 1). Specifically, in ciders, the relative concentration of esters increased 9- and ~12-fold on average in comparison with fruits and juices, respectively, due to the fermentation process. Additionally, alcohols are other chemical compounds were found in high concentration in ciders (4407.18 µg L^−1^ on average), ~6- and 4-fold, when compared to apple fruits and juices, respectively (773.66 and 1157.17 µg L^−1^ on average, respectively) (Figure 1).

In apple fruits, the VOCs of highest relative concentration were 2-hexenal (on average, 428.83 µg kg^−1^), 1-hexanol (403.70 µg kg^−1^), and α-farnesene (368.27 µg kg^−1^); in juices, they were 2-hexenal (on average, 663.13 µg L^−1^), 1-hexanol (515.70 µg L^−1^), and 3-methyl-1-butanol (367.07 µg L^−1^). In ciders, the VOCs of highest relative concentration were ethyl octanoate (on average, 3659.23 µg L^−1^), ethanol (2582.63 µg L^−1^), and 3-methyl-1-butanol (1439.60 µg L^−1^), as shown in Table 1.

**Figure 2:**
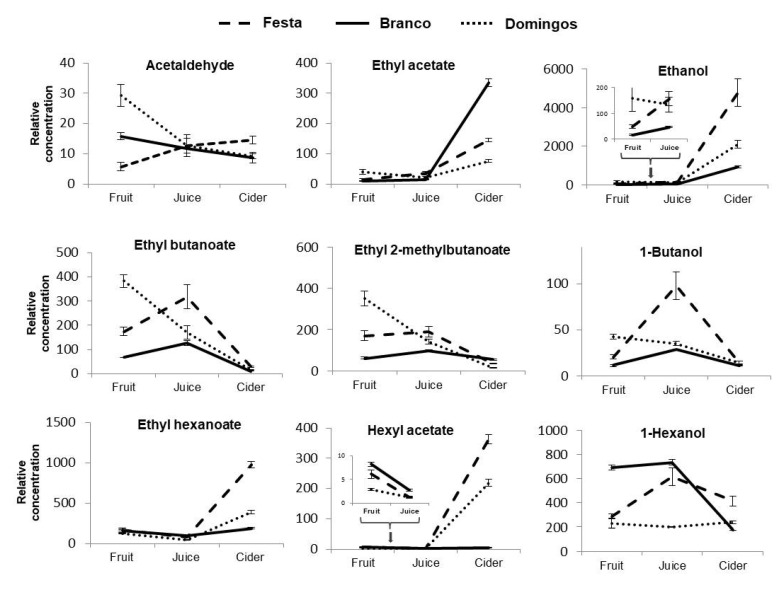
Relative concentration of volatile organic compounds (VOCs) common to all apple fruits (µg kg^−1^), juices, and ciders (µg L^−1^) (*n* = 3 for each data point).

Among 142 identified and semiquantified VOCs, only 9 (acetaldehyde (1), ethyl acetate (3), ethanol (8), ethyl butanoate (17), ethyl 2-methylbutanoate (20), 1-butanol (29), ethyl hexanoate (43), hexyl acetate (49) and 1-hexanol (67)) were common in all analysed matrices (fruit, juice, and cider) and in all apple-tested varieties (Festa, Branco, and Domingos) (Figure 2).

Among these 9 VOCs, acetaldehyde (1), ethyl butanoate (17), and ethyl 2-methylbutanoate (20) were higher in fruits than in juices and ciders, whereas ethyl acetate (3), ethanol (8), ethyl hexanoate (43), and hexyl acetate (49) were the largest VOCs detected in ciders. In the current study, there were statistical differences (p < 0.05) among the relative concentrations of these common VOCs according to different matrices and among apple varieties (Table 1). Nonetheless, in the case of acetaldehyde (1), there were no differences in the juices from three apple varieties. Similarly, when we compared apple fruits from 3 varieties for ethyl hexanoate (43), there were no statistically significant differences. Related to 1-hexanol (67), there were no statistical differences among fruits, juices, and ciders from the Domingos variety (Table 1 and Figure 2).

### 3.2. Contribution of Apple Matrices (Fruit, Juice, and Cider) on Volatile Profile

**Figure 3:**
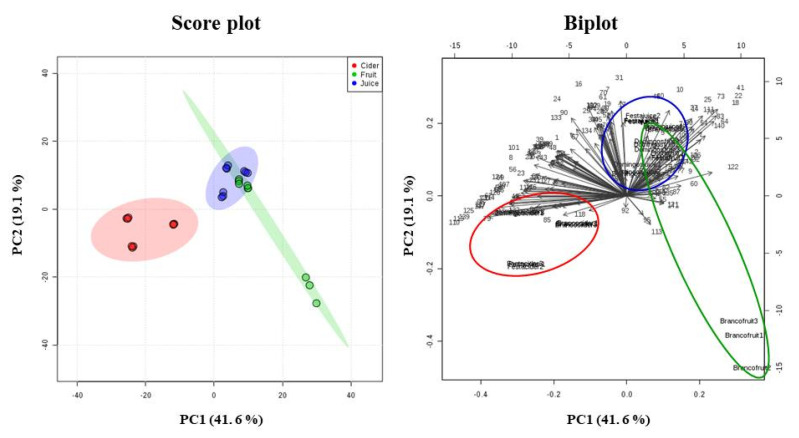
Principal-component analysis (PCA) of volatile profile of apple fruits, juices, and ciders based on apple variety (*n* = 3 for each data point). Peak number attribution shown in Table 1.

As stated above, this study allowed for identifying common VOCs among 3 matrices studied from 3 different varieties (Figure 2), and other specifics for each commodity (Table 1). In order to differentiate apple-fruit, -juice, and -cider samples by volatilomic profile, principal-component analysis (PCA) was performed (Figure 3).

**Figure 4:**
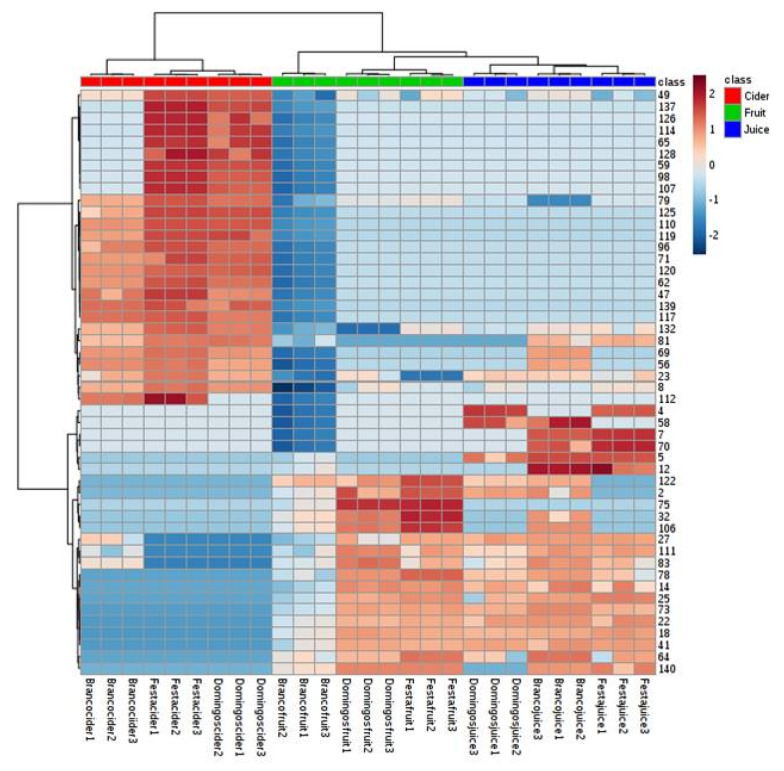
Hierarchical-cluster analysis (HCA). Heat-maps of putative markers identified in apple fruits, juices, and ciders generated by Euclidean distance through Ward agglomerative method (peak number attribution shown in Table 1).

Thus, effective separation according to apple matrices was achieved. The closeness of the samples on the PCA score plot indicated a similar volatile profile, and the PCA biplot showed the relationship between loadings (VOCs) and variables (fruits, juices, and ciders). The variance of PC1 and PC2 was 41.6% and 19.1%, respectively, representing 60.7% of the total variability of data, allowing for good differentiation among apple fruits, juices, and ciders. Combining the variable-importance-in-projection (VIP) values from PLS-DA higher than 1.6 (data not shown), 15 VOCs were selected as putative markers for discrimination among apple fruits, juices, and ciders. These putative markers were 2-propanol (5), toluene (18), hexanal (22), propyl butanoate (25), 2-hexenal (41), styrene (47), 2-hexen-1-ol isomer (73), ethyl octanoate (79), ethyl nonanoate (96), ethyl decanoate (110), diethyl butanedioate (117), ethyl 9-decenoate (119), 3-(methylthio)-1-propanol (120), 2-phenylethyl acetate (125) and octanoic acid (139). In addition, heat-map clustering based on significant features from ANOVA and Tukey’s post hoc test was carried out to display data distribution and to compare the respective relatively quantified levels of VOCs throughout the apple matrices (Figure 4). 

More specifically, in apple fruits, esters such as butyl butanoate (42), hexyl propanoate (66), butyl hexanoate (75) were related to sweet fruity green apples and over-ripe fruit as odour attributes. One ketone (1-octen-3-one (55)), 1 terpenoid as α-farnesene isomer ((Z–E)-α-farnesene) (121), and 1 lactone (2-hydroxy-γ-butyrolactone (141)) were only detected in fruit samples in at least 2 varieties, not identifying either in juices or in ciders.

Regarding juices, several VOCs were exclusively identified in this matrix, namely, butanal (4), 2,4,5-trimethyl-1,3-dioxolane (7), 1-penten-3-ol (28), 2-penten-1-ol (58), and 2-butoxyethanol (70) with pungent, horseradish, green vegetables, and a tropical-fruit odour description; these VOCs were not identified in either fruit or cider samples. 

The main chemical families of VOCs found in ciders that formed the fermentation bouquet were esters and alcohols, and aldehydes and ketones to a lesser extent (Figure 1). In this way, among esters, methyl octanoate (71), ethyl nonanoate (96), ethyl decanoate (110), diethyl butanedioate (117), ethyl 9-decenoate (119), and 2-phenylethyl acetate (125) were identified in all cider samples from the 3 investigated varieties (Table 1). Acids could also be important odour compounds in the ciders, such as octanoic acid (139), with a sweaty cheese aroma that was only found in ciders, but not in fruits or juices. However, this VOC was semiquantified below its odour threshold (OT ~3000 µg L^−1^). Among alcohols detected in ciders, 2 (3-methyl-1-pentanol (62) and 3-(methylthio)-1-propanol (120)) were identified in the ciders from the 3 varieties. Regarding the terpenoids detected only in ciders, β-damascenone (126), characterized by a woody, sweet, fruity, green, and floral aroma, was found in 2 of the 3 studied varieties, ranging between 13.2 and 17.8 µg L^−1^. Additionally, in the current study, styrene (47) was only identified in ciders and was quantified for the first time. It is a terpenoid with sweet, balsamic, floral, and plastic odour attributes, and it has only been detected in ciders from all studied varieties, the Festa variety being the samples with the highest relative concentration (67.5 µg L^−1^).

On the other hand, there were several VOCs that were not identified in cider samples from the three varieties (Table 1). For example, α-farnesene (122) was identified in apple fruits and juices, but not detected in ciders. Moreover, toluene (18) with the sweet aroma is another VOC found in apple fruits and juices, but not detected in cider samples. Additionally, some VOCs, such as hexanal (22) and 2-hexenal (41), found in apple fruits and juices from the three different varieties, were not identified in ciders (Table 1). Likewise, the ketone family were decreased in ciders in comparison with in the fruits and juices (Figure 1). Four ketones (2-propanone (2), 3-octanone (46), 6-methyl-5-hepten-2-one (64) and 1,3-dihydroxy-2-propanone (140)) were not found in ciders, but they were identified in fruits and juices.

### 3.3. Impact of Apple Variety on Volatile Profile

Food-authenticity issues may be solved by the detection and eventual quantification of specific metabolites that are able to discriminate among specific varieties, as shown in Table 2. In this way, for example, in apple-fruit samples, α-farnesene (122) was detected in all varieties, but (Z,E)-α-farnesene (121) was only identified in 2 varieties (Festa and Branco). The same applied in the case of linalool (95), which in the current study was only identified in Branco fruit samples. Furthermore, another terpenoid (estragole (113) with sweet, phenolic, anise, spicy, green, herbal, and minty aroma descriptors) was only detected in apple fruits from the Branco variety, as can be seen in Table 1 and Table 2. Thus, these VOCs could serve as authenticity indicators to verify apple-fruit variety. Another VOC only detected in the Branco variety (fruit and juice) was 2-nonenal (94). The Domingos apple sample was the variety with more unique VOCs in comparison with those in the other varieties (Festa and Branco) (Table 2). In fact, benzothiazole (134) was a unique VOC to the Domingos apple juice and regarding the VOCs that were only present in Domingos ciders, such as pentyl acetate (33), decanal (89), citronellol (123), geranylcetone (129), or nerolidol (138) (Table 2). This find provides us with a clear overview of the importance of the selection of apple varieties as a crucial factor for the cider-making process to obtain a cider of good sensory and quality properties.

## 4. Discussion

There are serious economic and quality reasons to certify the authenticity of varieties used in different food commodities. Moreover, as food processing progresses, for example, from apple fruits to ciders, it becomes extremely difficult to distinguish between varieties [13]. In this respect, a volatilomic pattern may be a useful tool to discriminate between food commodities and varieties. The main precursors of VOCs in apple fruits are fatty acids that are catabolized through β-oxidation and the lipoxygenase (LOX) pathway, which produce aldehydes, alcohols, and esters. Among these, aldehydes are predominant in immature apples, whereas alcohols and esters prevail in ripe/over-ripe fruits [22]. Regarding the different investigated variables (such as apple variety, ripening stage, and yeast strain), apple variety proved to be the primary attribute influencing the quality and aroma properties of apple ciders [23]. Three sources of VOCs in ciders, namely, apple juices, yeast, and yeast metabolism, were reported [4]. In the current study, the main chemical families of VOCs found in ciders that conferred the fermentation bouquet were esters and alcohols, and aldehydes and ketones to a lesser extent, as previously reported [24]. Regarding the different chemical families of VOCs, esters positively contribute to the aroma profile of ciders, bringing fruity and floral sensory properties [25]. More specifically, ethyl hexanoate (sweet, fruity, pineapple, waxy, fatty, estery, green, and banana odour descriptions) was reported as a VOC that increases in ciders in comparison with apple juices [26]. This VOC was associated with the fermentative process and the involved yeast strains [27], and, together with ethyl decanoate and ethyl octanoate, determines fruity and floral aromas in fermented fruit beverages [9]. However, there are other VOCs, such as 2-hexenal and 1-hexanol, which were described as the main contributors to the green odour of apple fruits and juices [4,28].

Regarding the fermentation process of apple juices, Antón et al. [25] found 3-methyl-1-pentanol to be a VOC that increases its concentration in cider samples from spontaneous fermentation in comparison with ciders from commercial *Saccharomyces cerevisiae*. This might be justified by yeast species associated with the spontaneous fermentation of both *Saccharomyces* and non-*Saccharomyces* yeasts (*Hanseniaspora* genus and *Metschnikowia pulcherrima*) that could affect concentrations of VOCs in ciders [29]. Styrene is another VOC reported in apple brandy and cider, with odour threshold values ranging between 3.6 to 80 µg L^−1^ [30], and in apple fruits [31]. The formation of this VOC may be because high cinnamic acid content and yeast pitching rate, in combination with open fermentation management, cause quick and increased styrene formation during fermentation, as was previously reported for wheat beer [32]. Thus, styrene may be used as an important indicator to monitor the cider-making process (as well as in beers) and management with food authentication purposes. In contrast, other VOCs were not detected in cider samples, such as toluene, which may be due to the toluene degradation pathway of *S. cerevisiae* (M00418 KEGG pathway) producing benzyl alcohol and benzaldehyde. Both VOCs were found in the current study in ciders. Recently, toluene was reported in apples for the first time [31] and was also identified in apple-juice samples from Madeira as a putative biomarker for the discrimination of the geographical origin of apple juices [13]. Furthermore, the conversion mechanism of benzyl alcohol to toluene in fruit juices was also recently reported [33].

On the other hand, there are VOCs that allow for distinguishing apple varieties. In this sense, (Z,E)-α-farnesene was only identified in 2 varieties (Festa and Branco) about 100 times less than another isomer ((E,E)-α-farnesene) [34]. In this context, in a previous study, (Z,E)-α-farnesene was able to differentiate banana plant cultivars since this VOC was detected in the Pacific plantain cultivar, but not identified in Cavendish cultivar, whereas (E,E)-α-farnesene was detected in both banana cultivars [35]. Hence, (Z,E)-α-farnesene might be used as a putative marker to discriminate apple-fruit varieties for food authenticity purposes. The same applies in the case of linalool, which, in the banana study mentioned above, was only detected in Pacific plantain and, in the current study, was only identified in Branco fruit samples; this VOC showed insect- and disease-control properties [35], with benefits for the quality of apple fruits. Both linalool and estragole could differentiate basil varieties [36]. In addition, 2-nonenal, with waxy and fatty aroma descriptors, was previously used to distinguish among 10 different fresh jujube varieties by HS-SPME/GC–MS coupled with E-nose [37]. Benzothiazole was also identified as a putative marker for distinguishing apple varieties from Madeira in a previous study on apple juices recently carried out by our research group [13]. 

## 5. Conclusions

HS–SPME/GC–MS combined with chemometric tools was successfully applied to explore the similarities and differences among apple fruits, juices, and ciders from different apple varieties (Festa, Branco, Domingos). A total of 142 VOCs belonging to different chemical families were identified, namely, 58 esters, 34 alcohols, 19 aldehydes, 10 ketones, 8 terpenoids, 7 acids, 3 sulphur compounds, 1 dioxolane, 1 lactone, and 1 aromatic hydrocarbon. From these, only 9 VOCs were detected in all analysed matrices (fruit, juice, and cider) and in all apple-tested varieties (Festa, Branco, and Domingos). Moreover, remarkable difference in terms of the qualitative and semiquantitative profiles was observed, which indicated that apple variety has a significant effect on the volatile profile. Esters and alcohols were the dominant chemical families, contributing 48.81%, 56.75%, and 94.04% on average for the total volatile profile of apple fruits, juices, and ciders, respectively. In qualitative terms, butyl butanoate (42), 1-octen-3-one (55), hexyl propanoate (66), butyl hexanoate (75), α-farnesene (122) and 2-hydroxy-γ-butyrolactone (141) were only detected in apple fruits, whereas butanal (4), 2,4,5-trimethyl-1,3-dioxolane, (7) 1-penten-3-ol (28), 2-penten-1-ol (58), and 2-butoxyethanol (70) were found in juices. On the other hand, methyl octanoate (38), styrene (47), 3-methyl-1-pentanol (62), ethyl 9-decenoate (119), and octanoic acid (139) were detected in all ciders. Moreover, there were VOCs that were of unique variety, such as benzyl alcohol (130) for the Festa, linalool (95) and estragole (113) for the Branco, and decanal (89) and benzothiazole (134) for the Domingos apple varieties. Accordingly, VOCs could be used as authenticity indicators to classify fruits, juices, and ciders according to apple variety, providing local producers with multiple benefits and legal protection against the misuse of the products.

## Figures and Tables

**Table 1:** Relative concentration of volatile organic compounds (VOCs) identified in apple fruits (µg kg^−1^), juices (µg L^−1^), and ciders (µg L^−1^) of different varieties (Festa, Branco, and Domingos) by headspace solid-phase microextraction gas chromatography–quadrupole mass spectroscopy (HS-SPME/GC–qMS).

					Festa			Branco			Domingos		
Id	RT ^a^	Compound Name	KI ^b^	KI ^c^	Fruit	Juice	Cider	Fruit	Juice	Cider	Fruit	Juice	Cider
1	4.7	Acetaldehyde (A) ^d^	629	690	5.7 ± 1.4 Bc	12.7 ± 2.4 An.s	14.5 ± 1.3 Aa	15.8 ± 1.1 Ab	11.0 ± 0.3 B n.s	8.7 ± 1.7 Bb	29.3 ± 3.7 Aa	12.7 ± 3.6 B n.s	9.1 ± 0.8 Bb
2	5.9	2-Propanone (K)	816	814	4.6 ± 0.6 b	-	-	11.9 ± 1.8 Aa	1.1 ± 0.4 n.s	-	6.0 ± 0.5 Ab	0.9 ± 0.1 B n.s	-
3	6.9	Ethyl acetate (E) ^d^	874	907	13.2 ± 3.4 Cb	35.0 ± 6.4 Ba	145.4 ± 5.7 Ab	8.7 ± 0.9 Bb	13.1 ± 1.0 Bb	334.7 ± 13.2 Aa	40.2 ± 8.2 Ba	20.9 ± 3.1 Cb	75.8 ± 4.7 Ac
4	6.9	Butanal (A)	876	878	-	0.8 ± 0.1 b	-	-	-	-	-	1.8 ± 0.3 a	-
5	7.1	2-Propanol (AL)	884	885	-	2.9 ± 0.1 b	-	2.6 ± 0.5 n.s	3.5 ± 0.2 n.sa	-	-	1.3 ± 0.04 c	-
6	7.5	2-Methylbutanal (A) ^d^	906	914	-	4.1 ± 0.5	-	-	-	-	-	-	-
7	7.6	2,4,5-Trimethyl-1,3-dioxolane (D)	907	967	-	3.9 ± 0.3 a	-	-	1.5 ± 0.07 b	-	-	-	-
8	7.9	Ethanol (AL) ^d^	920	929	49.5 ± 7.4 Bb	156.7 ± 27.3 Ba	4798.6 ± 714.6 Aa	17.1 ± 3.3 Bb	47.6 ± 2.3 Bb	939.7 ± 50.5 Ab	158.8 ± 28.3 Ba	133.5 ± 32.8 Ba	2009.6 ± 207.4 Ab
9	8.6	Pentanal (A) ^d^	943	935	16.5 ± 2.6 a	-	-	19.4 ± 3.6 A n.s	2.3 ± 0.2 Ba	-	-	1.2 ± 0.2 b	-
10	8.7	Ethyl propanoate (E) ^d^	949	959	27.9 ± 3.5 Ba	99.0 ± 18.6 Aa	-	3.3 ± 0.1 Cb	6.1 ± 0.5 Bb	20.0 ± 0.6 A	24.5 ± 4.5 Aa	8.1 ± 1.3 Bb	-
11	9.1	Ethyl 2-methylpropanoate (E)	959	955	-	6.6 ± 0.7 a	-	-	1.7 ± 0.1 Bb	4.2 ± 0.1 A	-	-	-
12	9.6	Methyl butanoate (E)	976	982	-	2.0 ± 0.2 a	-	1.9 ± 0.4 A	1.2 ± 0.1 Bb	-	-	-	-
13	10.6	Isobutyl acetate (E)	1006	1015	-	-	-	-	-	1.9 ± 0.01	-	-	-
14	10.7	Methyl 2-methylbutanoate (E)	1007	1033	2.0 ± 0.4 n.sa	2.7 ± 0.4 n.sa	-	4.0 ± 0.8 n.sa	3.4 ± 0.3 n.sa	-	2.3 ± 0.4 Ab	1.0 ± 0.2 Bb	-
15	10.8	1-Penten-3-one (K)	1011	1024	-	-	-	7.7 ± 0.5 A	2.8 ± 0.2 B	-	-	-	-
16	11.4	1-Propanol (AL) ^d^	1028	1037	2.3 ± 0.3 Bb	30.1 ± 6.9 Aa	-	-	1.2 ± 0.01 Bb	4.9 ± 0.12 Aa	3.0 ± 0.06 Aa	-	1.5 ± 0.2 Bb
17	11.6	Ethyl butanoate (E) ^d^	1031	1040	174.7 ± 17.6 Bb	318.0 ± 49.6 Aa	29.6 ± 2.9 Ca	68.1 ± 0.1 Bc	127.2 ± 9.4 Ab	11.1 ± 0.3 Cc	383.1 ± 26.3 Aa	170.1 ± 27.2 Bb	16.9 ± 1.5 Cb
18	11.9	Toluene (ArHC)	1039	1040	48.1 ± 7.4 Bc	90.1 ± 17.1 Aa	-	214.8 ± 12.5 Aa	46.9 ± 2.5 Bb	-	80.2 ± 12.3 Ab	29.3 ± 0.5 Bb	-
19	11.9	Propyl propanoate (E)	1039	1056	1.6 ± 0.01 B	5.8 ± 0.9 A	-	-	-	-	-	-	-
20	12.3	Ethyl 2-methylbutanoate (E)	1049	1050	170.2 ± 24.8 Ab	190.0 ± 26.1 Aa	34.2 ± 1.3 Bb	59.6 ± 6.8 Bc	96.7 ± 6.5 Ac	54.0 ± 2.5 Ba	351.1 ± 36.4 Aa	141.1 ± 11.9 Bb	14.6 ± 0.6 Cc
21	12.6	Butyl acetate (E)	1056	1075	-	-	-	-	-	0.9 ± 0.1 b	-	0.5 ± 0.1 B	3.1 ± 0.1 Aa
22	13.4	Hexanal (A)	1076	1080	116.3 ± 6.4 Bb	426.5 ± 55.2 Aa	-	275.0 ± 42.0 n.sa	294.0 ± 14.7 n.sb	-	69.4 ± 7.9 Bb	263.0 ± 33.8 Ab	-
23	13.8	2-Methyl-1-propanol (AL) ^d^	1085	1097	-	15.9 ± 2.33 Ba	187.5 ± 16.32 Aa	5.8 ± 1.9 Cn.s	9.5 ± 1.2 Bb	34.3 ± 1.0 Ab	6.1 ± 1.4 Bn.s	10.4 ± 3.0 Bab	34.9 ± 3.3 Ab
24	15.1	2-Methyl-1-butyl acetate (E)	1112	1145	-	2.6 ± 0.39 a	-	-	0.7 ± 0.1 b	-	1.1 ± 0.2 B	1.8 ± 0.5 Ba	221.1 ± 6.4 A
25	15.6	Propyl butanoate (E)	1121	1135	10.5 ± 0.9 Ba	16.2 ± 1.7 Aa	-	11.3 ± 1.8 Aa	7.1 ± 0.4 Bb	-	5.1 ± 1.0 Ab	3.0 ± 0.5 Bc	-
26	15.8	2-Pentenal isomer (A)	1126	1131	4.9 ± 0.7 b	-	-	12.2 ± 2.0 Aa	2.3 ± 0.05 B	-	0.7 ± 0.1 c	-	-
27	16.2	Ethyl pentanoate (E)	1133	1138	6.9 ± 0.6 n.sb	8.2 ± 0.6 n.s n.s	-	12.3 ± 2.0 Aa	9.7 ± 0.7 An.s	1.9 ± 0.1 B	5.9 ± 1.1 n.sb	7.5 ± 2.2 n.sn.s	-
28	16.2	1-Penten-3-ol (AL)	1134	1176	-	-	-	-	1.7 ± 0.2 a	-	-	0.8 ± 0.1 b	-
29	16.3	1-Butanol (AL) ^d^	1136	1145	20.9 ± 2.1 Bb	98.0 ± 15.0 Aa	14.5 ± 1.2 Ba	12.5 ± 2.1 Bc	28.7 ± 1.1 Ab	11.4 ± 0.3 Bb	43.0 ± 2.6 Aa	35.5 ± 2.3 Bb	14.7 ± 1.4 Ca
30	16.5	Propyl-2-methylbutanoate (E)	1139	1150	13.9 ± 0.8 ab	-	-	16.6 ± 3.5 a	-	-	9.3 ± 1.3 Ab	5.5 ± 1.1 B	0.9 ± 0.1 C
31	17.5	Ethyl-2-butenoate (E)	1157	1152	-	3.7 ± 0.6 a	-	-	1.1 ± 0.1 b	-	1.9 ± 0.2 n.s	2.1 ± 0.4 n.sb	-
32	18.0	β-Myrcene (T)	1165	1167	17.9 ± 1.2 a	-	-	15.1 ± 1.3 Ab	1.2 ± 0.1 B	-	2.1 ± 0.3 c	-	-
33	18.3	Pentyl acetate (E)	1169	1177	-	-	-	-	-	-	-	-	1.3 ± 0.04
34	18.3	Butyl 2-methylbutanoate (E)	1170	1228	-	-	-	-	-	-	1.0 ± 0.2	-	-
35	18.5	2-Methylpropyl-2-methylbutanoate (E)	1173	1171	-	-	-	2.7 ± 0.03 A	1.6 ± 0.1 B	-	-	-	-
36	18.6	2-Heptanone (K)	1174	1185	-	-	-	-	-	0.8 ± 0.1 a	-	-	0.6 ± 0.02 b
37	18.6	Heptanal (A)	1175	1186	5.8 ± 0.2 b	-	-	7.8 ± 0.6 Aa	1.4 ± 0.1 Ba	-	-	0.8 ± 0.1 b	-
38	18.8	Methyl hexanoate (E)	1178	1190	1.9 ± 0.1 n.s b	1.7 ± 0.1 n.sb	-	10.3 ± 1.0 Aa	2.2 ± 0.2 Ba	0.5 ± 0.03 Cn.s	1.1 ± 0.2 Ab	0.4 ± 0.05 Bc	0.6 ± 0.1 Bn.s
39	19.1	Pentyl propanoate (E)	1184	1192	-	1.1 ± 0.1	-	-	-	1.6 ± 0.1 a	-	-	1.2 ± 0.1 b
40	19.6	3-Methyl-1-butanol (AL)	1191	1207	132.0 ± 15.3 Bb	725.0 ± 120.9 Aa	-	103.8 ± 4.9 Bb	178.8 ± 7.6 Ab	-	276.0 ± 18.2 Ba	197.4 ± 13.9 Bb	1439.6 ± 108.9 A
41	20.4	2-Hexenal (A) ^d^	1204	1220	225.7 ± 21.0 Bc	602.2 ± 62.4 Ab	-	678.2 ± 4.5 Ba	929.9 ± 43.0 Aa	-	382.6 ± 38.4 n.sb	457.3 ± 80.8 n.sb	-
42	20.5	Butyl butanoate (E)	1205	1223	2.5 ± 0.1 b	-	-	3.0 ± 0.1 a	-	-	-	-	-
43	21.5	Ethyl hexanoate (E) ^d^	1224	1220	162.6 ± 24.5 Bn.s	78.3 ± 3.7 Cb	975.8 ± 36.7 Aa	160.6 ± 10.6 Bn.s	96.1 ± 6.1 Ca	188.8 ± 8.7 Ac	124.5 ± 5.6 Bn.s	40.8 ± 3.4 Cc	382.3 ± 22.0 Ab
44	21.5	Ethyl 2-methyl-2-butenoate (E)	1226	1229	-	-	-	-	-	-	3.7 ± 0.6	-	-
45	22.0	1-Pentanol (AL)	1234	1253	7.0 ± 0.8 Bb	20.6 ± 3.1 Aa	-	9.7 ± 0.5 Ba	10.8 ± 0.3 Ab	2.4 ± 0.1 Cn.s	9.4 ± 0.6 Aa	6.2 ± 0.2 Bc	3.6 ± 0.8 Cn.s
46	22.6	3-Octanone (K)	1244	1251	2.3 ± 0.4 n.sb	2.0 ± 0.1 n.sb	-	4.3 ± 0.5 Aa	2.2 ± 0.1 Ba	-	-	-	-
47	22.7	Styrene (T)	1246	1241	-	-	67.5 ± 4.7 a	-	-	8.2 ± 0.5 a	-	-	3.7 ± 0.5 b
48	23.4	2-Methylbutyl butanoate (E)	1258	1268	2.5 ± 0.5 Ba	-	13.1 ± 0.8 Aa	-	-	-	0.9 ± 0.1 n.sb	1.0 ± 0.1 n.s	1.1 ± 0.05 n.sb
49	23.5	Hexyl acetate (E) ^d^	1260	1270	6.1 ± 0.9 Bb	1.2 ± 0.2 Bb	363.8 ± 15.1 Aa	8.2 ± 0.5 Aa	2.7 ± 0.2 Ca	4.8 ± 0.2 Bc	2.9 ± 0.2 Bc	1.2 ± 0.1 Bb	218.7 ± 12.3 Ab
50	23.8	Ethyl 5-hexenoate (E)	1265	1269	2.3 ± 0.4 b	-	-	4.5 ± 0.7 Aa	3.3 ± 0.00 B	-	-	-	4.6 ± 0.2
51	24.1	Amyl isovalerate (E)	1270	1285	-	1.3 ± 0.03 a	-	8.0 ± 1.0 A	1.2 ± 0.05 Bb	-	-	-	-
52	24.1	3-Methyl-2-methylbutyl butanoate (E)	1271	1283	1.4 ± 0.1 b	-	-	-	-	0.7 ± 0.01	4.7 ± 0.5 Ba	8.1 ± 1.5 A	-
53	24.4	Octanal (A)	1274	1286	4.1 ± 0.7 a	-	-	5.0 ± 0.5 a	-	-	0.8 ± 0.01 Bb	1.5 ± 0.05 A	0.8 ± 0.15 B
54	24.9	1-Hydroxy-2-propanone (K)	1283	1284	15.9 ± 0.8 Ab	9.1 ± 2.5 Bb	-	42.5 ± 16.1 Aa	13.9 ± 1.4 Ba	0.5 ± 0.1 B	21.3 ± 2.1 ab	-	-
55	25.0	1-Octen-3-one (K)	1284	1299	4.9 ± 0.9 n.s	-	-	5.3 ± 1.0 n.s	-	-	-	-	-
56	25.2	Ethyl 3-hexenoate (E)	1287	1292	-	-	2.6 ± 0.4 a	-	0.6 ± 0.1 B	1.4 ± 0.02 Ab	-	-	0.6 ± 0.1 c
57	25.2	4-Methyl-1-pentanol (AL)	1288	1299	-	-	5.1 ± 0.9	-	-	-	-	-	-
58	25.5	2-Penten-1-ol isomer (AL)	1292	1301	-	-	-	-	4.6 ± 0.8 a	-	-	1.3 ± 0.1 b	-
59	25.6	3-Hexenyl acetate isomer (E)	1294	1311	-	-	8.8 ± 0.5 a	-	-	-	-	-	3.8 ± 0.3 b
60	26.0	2-Heptenal isomer (A)	1300	1331	34.3 ± 3.5 n.s	-	-	39.0 ± 1.4 An.s	4.5 ± 0.1 B	-	-	-	-
61	26.2	Propyl hexanoate (E)	1305	1324	-	3.1 ± 0.1	-	-	-	-	2.1 ± 0.02	-	-
62	26.4	3-Methyl-1-pentanol (AL)	1307	1325	-	-	8.0 ± 1.4 a	-	-	0.9 ± 0.1 b	-	-	3.1 ± 0.2 b
63	26.8	Ethyl-2-hydroxypropanoate (E)	1316	1342	-	-	-	-	-	53.2 ± 2.5	-	-	-
64	27.0	6-Methyl-5-hepten-2-one (K)	1319	1340	37.6 ± 2.4 Ab	9.5 ± 0.9 Bb	-	171.2 ± 23.0 Aa	39.8 ± 2.1 Ba	-	4.0 ± 0.02 Ac	2.1 ± 0.1 Bc	-
65	27.3	Ethyl heptanoate (E)	1324	1337	-	-	12.6 ± 2.1 n.s	-	-	-	-	-	10.8 ± 1.1 n.s
66	27.4	Hexyl propanoate (E)	1325	1330	4.3 ± 0.2 a	-	-	3.9 ± 0.2 b	-	-	-	-	-
67	27.9	1-Hexanol (AL)	1335	1354	289.4 ± 17.2 Cb	614.0 ± 70.9 Ab	412.7 ± 41.1 Ba	692.6 ± 20.3 Aa	732.9 ± 24.6 Aa	174.3 ± 1.1 Bc	229.1 ± 37.8 n.sb	200.2 ± 3.7 n.sc	238.6 ± 10.6 n.sb
68	28.4	3-Hexen-1-ol (AL)	1344	1357	-	-	-	-	-	0.8 ± 0.02 b	1.7 ± 0.2B	-	6.9 ± 0.8 Aa
69	29.5	3-Hexen-1-ol isomer (AL)	1364	1388	-	-	19.9 ± 1.7 a	-	4.0 ± 0.5 B	5.1 ± 0.1 Ab	-	-	4.9 ± 0.7 b
70	30.2	2-Butoxyethanol (AL)	1375	1391	-	3.2 ± 0.3 a	-	-	0.9 ± 0.1 b	-	-	-	-
71	30.3	Methyl octanoate (E)	1376	1378	-	-	21.4 ± 0.9 a	-	-	1.2 ± 0.1 c	-	-	8.9 ± 1.1 b
72	30.3	Nonanal (A)	1377	1369	4.5 ± 0.3 Bb	-	7.7 ± 0.6 Aa	6.4 ± 1.0 Aa	-	0.3 ± 0.03 Bc	3.3 ± 0.1 n.sa	3.8 ± 0.8 n.s	3.0 ± 0.6 n.sb
73	30.7	2-Hexen-1-ol isomer (AL)	1383	1410	27.0 ± 4.8 Aa	13.5 ± 1.74 Bb	-	31.6 ± 2.6 Ba	61.4 ± 1.6 Aa	-	23. ± 4.15 Aa	14.8 ± 1.9 Bb	-
74	30.9	5-Hexen-1-ol (AL)	1388	1394	5.8 ± 1.1 Ba	17.9 ± 2.1Aa	-	4.8 ± 0.6 Ba	6.3 ± 0.1 Ab	-	-	-	-
75	31.5	Butyl hexanoate (E)	1397	1403	2.5 ± 0.3 n.s	-	-	2.8 ± 0.6n.s	-	-	2.0 ± 0.02 n.s	-	-
76	31.6	Hexyl 2-methylpropanoate (E)	1398	1339	9.6 ± 0.7	-	-	-	-	-	-	-	-
77	31.7	Hexyl butanoate (E)	1400	1419	-	-	-	13.2 ± 2.3 Aa	7.5 ± 0.5 B	-	1.7 ± 0.07 b	-	-
78	32.4	Hexyl 2-methylbutanoate (E)	1414	1431	23.1 ± 1.4 Ab	1.4 ± 0.1 Bb	-	27.6 ± 1.7 Aa	5.5 ± 0.6 Ba	-	6.4 ± 0.01 Ac	1.3 ± 0.2 B	-
79	32.7	Ethyl octanoate (E) ^d^	1419	1436	7.5 ± Ba	2.4 ± 0.05 Ba	8039.1 ± 578.3 Aa	7.1 ± 1.0 Ba	-	241.4 ± 15.7Ac	4.1 ± 0.3 Bb	1.3 ± 0.1 Bb	2697.2 ± 350.3 Ab
80	33.3	Acetic acid (AC)	1431	1447	58.5 ± 9.9 Ab	24.6 ± 6.5 Bn.s	-	133.6 ± 52.0 Aa	26.9 ± 6.1 Bn.s	11.2 ± 0.6 B	67.0 ± 2.3 b	-	-
81	33.5	1-Heptanol (AL) ^d^	1434	1460	-	2.9 ± 0.6 Bn.s	14.8 ± 1.2 Aa	9.3 ± 1.3 A	2.7 ± 0.2 Bn.s	1.8 ± 0.1 Bb	-	-	16.2 ± 1.8 b
82	33.6	2,4-Heptadienal isomer (A)	1436	1497	-	-	-	7.2 ± 0.4 A	3.4 ± 0.3 B	-	-	-	-
83	33.9	2-Furfural (A)	1441	1474	28.3 ± 2.5 Ab	21.2 ± 3.7 Bb	-	190.1 ± 44.4 Aa	85.8 ± 20.3 Ba	3.2 ± 0.3 C	202.3 ± 54.1 Aa	6.7 ± 1.7 Bb	-
84	33.9	6-Methyl-5-hepten-2-ol (AL)	1441	1468	-	-	-	-	-	-	-	-	10.6 ± 0.6
85	34.2	Isopentyl hexanoate (E)	1448	1453	4.1 ± 0.1 Bn.s	-	47.8 ± 1.1 Aa	4.3 ± 0.4 An.s	-	0.6 ± 0.04 Bc	4.2 ± 0.1 Bn.s	1.8 ± 0.4 C	6.5 ± 0.7 Ab
86	35.4	2-Ethyl 1-hexanol (AL) ^d^	1469	1484	16.1 ± 1.4 Aa	12.0 ± 0.8 Bn.s	14.0 ± 2.1 ABa	10.2 ± 1.0 Ab	10.8 ± 0.5 An.s	7.5 ± 0.1 Bb	-	-	-
87	36.0	Formic acid (AC)	1479	1487	-	-	-	24.4 ± 8.5 A	11.8 ± 1.1 B	-	-	-	-
88	36.1	2-Acetylfuran (K)	1481	1482	-	-	-	11.5 ± 2.9 A n.s	3.0 ± 0.4 B	-	16.3 ± 2.6 n.s	-	-
89	36.2	Decanal (A)	1483	1502	-	-	-	-	-	-	-	4.3 ± 0.6 B	6.7 ± 0.6 A
90	36.8	Ethyl 3-hydroxy-butanoate (E) ^d^	1492	1524	-	9.2 ± 0.7 a	-	-	-	-	-	3.5 ± 0.3 Ab	1.5 ± 0.2 B
91	37.0	2-Nonanol (AL)	1496	1528	-	-	-	-	-	-	-	-	1.3 ± 0.1
92	37.1	Benzaldehyde (A) ^d^	1498	1495	-	1.9 ± 0.3 B	20.2 ± 1.1 A	33.9 ± 3.0 A	2.7 ± 0.3 B	-	-	-	-
93	37.4	Dihydro-2-methyl-3(2H)-thiophenone (SC)	1504	1510	-	-	-	-	-	-	-	-	6.7 ±0.6
94	37.5	2-Nonenal isomer (A)	1506	1530	-	-	-	2.8 ± 0.3 A	0.7 ± 0.1 B	-	-	-	-
95	37.9	Linalool (AL)	1514	1554	-	-	-	2.3 ± 0.2	-	-	-	-	-
96	38.3	Ethyl nonanoate (E)	1522	1528	-	-	22.5 ± 1.2 a	-	-	4.1 ± 0.2 c	-	-	8.3 ± 0.3 b
97	39.0	1-Octanol (AL) ^d^	1535	1526	3.0 ± 0.4 Bns	-	24.0 ± 2.0 Aa	3.3 ± 0.5 An.s	1.1 ± 0.05 B	2.8 ± 0.1 Ab	-	-	5.3 ± 0.1 b
98	39.4	Isobutyl caprylate (E)	1543	1561	-	-	7.8 ± 0.2 a	-	-	-	-	-	2.0 ± 0.2 b
99	39.4	Ethyl 3-methylthiopropionate (E)	1548	1560	-	-	-	-	-	-	-	-	1.4 ± 0.1
100	39.7	5-Methyl-2-furfural (A)	1549	1559	-	2.1 ± 0.7 b	-	24.5 ± 2.4 Aa	12.0 ± 3.2 Ba	-	6.0 ± 1.9 b	-	-
101	41.3	4-Carvomenthenol (AL)	1579	1598	-	2.7 ± 0.3 Ba	23.6 ± 2.1 Aa	-	0.9 ± 0.03 b	-	-	-	16.8 ± 1.3 b
102	41.4	5-Octen-1-ol isomer (AL)	1581	1610	-	2.1 ± 0.1 b	-	-	2.5 ± 0.1 Aa	0.8 ± 0.02 B	-	-	-
103	41.7	2-Isopropyl-2-methylanisole (AL)	1587	1611	-	-	-	-	-	-	-	-	2.1 ± 0.4
104	41.9	Butanoic acid (AC)	1590	1581	-	-	-	-	-	2.4 ± 0.1	-	-	-
105	41.9	2-(2-Ethoxyethoxy)ethanol (AL)	1590	1579	-	-	-	-	-	-	2.8 ± 0.3	-	-
106	42.2	Hexyl hexanoate (E)	1595	1599	9.7 ± 9.6 a	-	-	12.1 ± 1.1 Aa	1.1 ± 0.02 B	-	1.9 ± 0.3 b	-	-
107	42.3	Ethyl 2-furoate (E)	1597	1621	-	-	5.8 ± 0.2 a	-	-	-	-	-	1.4 ± 0.2 b
108	42.7	Allyl methyl sulphide (SC)	1605	-	-	-	-	-	-	-	-	-	1.4 ± 0.1
109	43.4	Phenylacetaldehyde (A)	1619	1624	-	18.8 ± 2.3	-	-	-	-	-	-	-
110	43.6	Ethyl decanoate (E) ^d^	1624	1636	-	-	1341.5 ± 22.5 a	-	-	47.3 ± 4.0 c	-	-	905.1 ± 55.4 b
111	43.9	2-Furanmethanol (AL)	1630	1623	8.8 ± 1.2 b	7.6 ± 2.1 a	-	60.9 ± 5.9 Aa	9.1 ± 2.6 Ba	0.4 ± 0.01 B	16.6 ± 1.6 Ab	1.4 ± 0.2 Bb	-
112	44.2	1-Nonanol (AL)	1637	1662	-	-	14.8 ± 0.6 a	-	-	0.7 ± 0.1 b	-	-	-
113	44.4	Estragole (T)	1640	1661	-	-	-	4.4 ± 0.4	-	-	-	-	-
114	44.6	Ethyl benzoate (E)	1645	1653	-	-	70.7 ± 5.8 a	-	-	-	-	-	40.0 ± 2.9 b
115	44.7	2-Methylbutanoic acid (AC)	1647	1674	-	3.9 ± 0.6 b	-	152.9 ± 5.3 Ba	181.6 ± 7.4Aa	62.7 ± 5.8 C	4.8 ± 0.4 b	-	-
116	44.8	3-Methylbutyl octanoate (E)	1647	1658	-	-	85.7 ± 0.8	-	-	-	-	-	-
117	44.9	Diethyl butanedioate (E)	1648	1679	-	-	24.9 ± 4.8 a	-	-	10.6 ± 1.0 b	-	-	6.5 ± 0.4 b
118	45.3	4-Methoxystyrene (T)	1658	1688	-	-	-	4.2 ± 0.1	-	-	-	-	2.2 ± 0.4
119	46.0	Ethyl-9-decenoate (E)	1672	1694	-	-	756.3 ± 26.0 b	-	-	13.5 ± 1.1 c	-	-	964.1 ± 106.1 a
120	46.8	3-(Methylthio)-1-propanol (AL)	1687	1686	-	-	8.4 ± 1.2 a	-	-	1.7 ± 0.1 b	-	-	8.8 ± 1.7 a
121	48.0	α-Farnesene isomer (T)	1710	1721	4.4 ± 0.8 b	-	-	12.8 ± 2.1 a	-	-	-	-	-
122	49.1	α-Farnesene (T)	1733	1725	260.6 ± 36.1 b	-	-	819.8 ± 162.3 Aa	9.4 ± 0.7 Ba	-	24.4 ± 3.9 Ab	2.7 ± 0.5 Bb	-
123	49.3	Citronellol (T)	1736	1754	-	-	-	-	-	-	-	-	5.2 ± 0.5
124	50.3	Ethyl benzeneacetate (E)	1756	1763	-	-	11.1 ± 1.2 b	-	-	3.7 ± 0.2 c	2.8 ± 0.1 B	3.3 ± 0.3 B	17.1 ± 1.4 Aa
125	51.7	2-Phenylethyl acetate (E)	1783	1785	-	-	166.0 ± 13.7 a	-	-	2.7 ± 0.1 c	-	-	106.2 ± 10.9 b
126	52.2	β-Damascenone (T)	1793	1806	-	-	17.8 ± 0.6 a	-	-	-	-	-	13.2 ± 1.2 b
127	53.1	Hexanoic acid (AC)	1815	1849	11.3 ± 1.5 Ba	6.1 ± 0.8 Bb	89.6 ± 4.7 Ab	12.6 ± 0.7 Ba	14.5 ± 1.0 Ba	46.7 ± 5.7 Ac	2.1 ± 0.2 Bb	-	119.0 ± 9.4 Aa
128	53.5	Ethyl dodecanoate (E)	1823	1847	-	-	51.7 ± 9.1 a	-	-	-	-	-	17.4 ± 1.9 b
129	53.7	Geranylacetone (K)	1828	1840	-	-	-	-	-	-	-	-	2.0 ± 0.2
130	54.2	Benzyl alcohol (AL)	1841	1822	-	-	6.9 ± 0.5	-	-	-	-	-	-
131	54.3	3-Methylbutyl pentadecanoate (E)	1844	-	-	-	10.4 ± 1.3	-	-	-	-	-	-
132	55.8	2-Phenylethanol (AL) ^d^	1879	1859	7.9 ± 1.1 Bb	13.9 ± 1.7 Ba	1400.8 ± 260.9 Aa	14.4 ± 2.1 Ba	9.8 ± 1.6 Bb	93.3 ± 7.6 Ac	-	1.6 ± 0.3 Bc	548.4 ± 46.2 Ab
133	57.3	Ethyl 3-hydroxyhexanoate (E)	1913	-	-	4.9 ± 0.7 b	-	-	-	-	-	2.6 ± 0.5 Bb	5.5 ± 0.7 A
134	58.0	Benzothiazole (SC)	1929	1952	-	-	-	-	-	-	-	2.3 ± 0.3	-
135	58.8	2,5-Furandicarboxaldehyde (A)	1948	1996	-	-	-	14.2 ± 0.7 b	-	-	24.5 ± 1.4 a	-	-
136	60.0	Isopentyl-phenyl acetate (E)	1976	1991	-	-	-	-	-	-	-	1.2 ± 0.2 B	4.0 ± 0.6 A
137	61.2	4-Ethyl 2-methoxyphenol (AL)	2000	2020	-	-	524.0 ± 35.2a	-	-	-	-	-	91.7 ± 16.2 b
138	61.6	Nerolidol (AL)	2002	2025	-	-	-	-	-	-	-	-	2.7 ± 0.3
139	62.6	Octanoic acid (AC)	2008	2050	-	-	508.1 ± 35.1 a	-	-	122.2 ± 9.9 c	-	-	238.2 ± 23.8 b
140	63.4	1,3-Dihydroxy-2-propanone (K)	2012	2068	16.6 ± 0.7 Bb	19.8 ± 0.2 Aa	-	84.3 ± 18.2 Aa	9.8 ± 0.1 Bb	-	16.5 ± 0.9 b	-	-
141	66.6	2-Hydroxy-γ-butyrolactone (L)	2028	-	-	-	-	26.6 ± 3.9 a	-	-	12.7 ± 1.1 b	-	-
142	69.7	Hexadecanoic acid (AC)	2043	2009	263.5 ± 23.7 a	-	-	49.9 ± 16.1 Ab	8.0 ± 0.6 B	-	-	-	-

(A), aldehyde; (AC), acid; (AL), alcohol; (ArHC), aromatic hydrocarbon; (D), dioxolane; (E), ester; (K), ketone; (L), lactone; (SC), sulphur compound; (T), terpenoid. ^a^ Retention time (min). ^b^ Kovats index relative to n-alkanes (C_8_ to C_20_) on a BP-20 capillary column. ^c^ Kovats index relative reported in the literature for equivalent capillary column [1,21] and databases available online (The Pherobase and Flavornet). ^d^ Identified using pure standards (at concentration of 2.94 µg L^−1^). -, not detected; n.s, not significant. Mean concentration of 3 replicates relative to internal standard (3-octanol). Data shown as mean ± SD. Different lowercase letters in a row are significantly different among varieties (Festa, Branco, and Domingos) of the same matrix; different uppercase letters in a row represent statistically significant differences among different matrices (fruit, juice, and cider) of the same variety obtained by one-way ANOVA and Tukey’s multiple test at *p* < 0.05 level.

**Table 2:** Volatile organic compounds (VOCs) identified only in specific apple varieties (Festa, Branco, and Domingos).

Festa	Branco	Domingos
Propyl propanoate (19) ^a^ (*F*, *J*) Hexyl 2-methylpropanoate (76) (*F*) Phenylacetaldehyde (109) (*J*) 3-Methylbutyl octanoate (116) (*C*) Benzyl alcohol (130) (*C*) 3-Methylbutyl pentadecanoate (131) (*C*)	2-Methylpropyl-2-methylbutanoate (35) (*F*, *J*) 2,4-Heptadienal isomer (A) (82) (*F*, *J*) Formic acid (87) (*F*, *J*) 2-Nonenal isomer (94) (*F*, *J*) Linalool (95) (*F*) Butanoic acid (104) (*C*) Estragole (113) (*F*)	Pentyl acetate (34) (*C*) Butyl 2-methylbutanoate (34) (*F*) Ethyl 2-methyl-2-butenoate (44) (*F*) 6-Methyl-5-hepten-2-ol (AL) (84) (*C*) Decanal (A) (89) (*J*, *C*) 2-Nonanol (AL) (91) (*C*) Dihydro-2-methyl-3(2H)-thiophenone (93) (*C*) Ethyl 3-methylthiopropionate (99) (*C*) 2-(2-Ethoxyethoxy)ethanol (105) (*F*) Allyl methyl sulphide (108) (*C*) Citronellol (123) (*C*) Geranylacetone (129) (*C*) Benzothiazole (134) (*J*) Isopentyl-phenyl acetate (136) (*J*, *C*) Nerolidol (138) (*C*)

(C), cider; (F), fruit; (J), juice. ^a^ Numbers in brackets match with numbers assigned for VOCs listed in Table 1.

## Supplementary Materials

The following are available online at https://www.mdpi.com/2304-8158/9/12/1830/s1. Figure S1. Total ion chromatograms obtained by HS-SPME/GC-qMS analysis of apple fruits, juices, and ciders of the different varieties (Festa, Branco, Domingos).

## References

[1] Ferreira L., Perestrelo R.M.D.S., Caldeira M.M.L., Câmara J.D.S. (2009). Characterization of volatile substances in apples from Rosaceae family by headspace solid-phase microextraction followed by GC-qMS. J. Sep. Sci..

[2] Holland D., Larkov O., Bar-Ya’Akov I., Bar E., Zax A., Brandeis E., Ravid U., Lewinsohn E. (2005). Developmental and Varietal Differences in Volatile Ester Formation and Acetyl-CoA: Alcohol Acetyl Transferase Activities in Apple (Malus domesticaBorkh.) Fruit. J. Agric. Food Chem..

[3] Guo J., Yue T., Yuan Y. (2012). Feature Selection and Recognition from Nonspecific Volatile Profiles for Discrimination of Apple Juices According to Variety and Geographical Origin. J. Food Sci..

[4] Rita R.-D., Zanda K., Daina K., Dalija S. (2011). Composition of aroma compounds in fermented apple juice: Effect of apple variety, fermentation temperature and inoculated yeast concentration. Procedia Food Sci..

[5] Wei J., Zhang Y., Qiu Y., Guo H., Ju H., Wang Y., Yuan Y., Yue T. (2020). Chemical composition, sensorial properties, and aroma-active compounds of ciders fermented with Hanseniaspora osmophila and Torulaspora quercuum in co- and sequential fermentations. Food Chem..

[6] Antón-Díaz M.J., Valles B.S., Alonso J.J.M., Fernández-García O., Lobo A.P. (2016). Impact of different techniques involving contact with lees on the volatile composition of cider. Food Chem..

[7] Lobo A.P., Antón-Díaz M.J., Alonso J.J.M., Valles B.S. (2016). Characterization of Spanish ciders by means of chemical and olfactometric profiles and chemometrics. Food Chem..

[8] Laaksonen O., Kuldjärv R., Paalme T., Virkki M., Yang B. (2017). Impact of apple cultivar, ripening stage, fermentation type and yeast strain on phenolic composition of apple ciders. Food Chem..

[9] Braga C.M., Zielinski A.A.F., Da Silva K.M., De Souza F.K.F., Pietrowski G.D.A.M., Couto M., Granato D., Wosiacki G., Nogueira A. (2013). Classification of juices and fermented beverages made from unripe, ripe and senescent apples based on the aromatic profile using chemometrics. Food Chem..

[10] Dos Santos T.P.M., Alberti A., Judacewski P., Zielinski A.A.F., Nogueira A. (2018). Effect of sulphur dioxide concentration added at different processing stages on volatile composition of ciders. J. Inst. Brew..

[11] Perestrelo R., Silva C.L., Silva P., Medina S., Pereira R., Câmara J.S. (2019). Untargeted fingerprinting of cider volatiles from different geographical regions by HS-SPME/GC-MS. Microchem. J..

[12] Xu Y., Fan W., Qian M.C. (2007). Characterization of Aroma Compounds in Apple Cider Using Solvent-Assisted Flavor Evaporation and Headspace Solid-Phase Microextraction. J. Agric. Food Chem..

[13] Medina S.F., Perestrelo R., Santos R., Pereira R., Câmara J.S. (2019). Differential volatile organic compounds signatures of apple juices from Madeira Island according to variety and geographical origin. Microchem. J..

[14] Nešpor J., Karabín M., Štulíková K., Dostálek P. (2019). An HS-SPME-GC-MS Method for Profiling Volatile Compounds as Related to Technology Used in Cider Production. Molecules.

[15] Blanpied G.D., Silsby K.J. (1992). Predicting Harvest Date Windows for Apples. Cornell Coop. Ext..

[16] Barrett D., Garcia E., Lamikanra O. (2002). Preservative Treatments for Fresh-cut Fruits and Vegetables. Fresh-Cut Fruits and Vegetables: Science, Technology and Market.

[17] Dool H.V.D., Kratz P.D. (1963). A generalization of the retention index system including linear temperature programmed gas—liquid partition chromatography. J. Chromatogr. A.

[18] Pereira V., Cacho J., Marques J.C. (2014). Volatile profile of Madeira wines submitted to traditional accelerated ageing. Food Chem..

[19] Worley B. (2012). Multivariate Analysis in Metabolomics. Curr. Metab..

[20] Chong J., Soufan O., Li C., Caraus I., Li S., Bourque G., Wishart D.S., Xia J. (2018). MetaboAnalyst 4.0: Towards more transparent and integrative metabolomics analysis. Nucleic Acids Res..

[21] Bianchi F., Careri M., Mangia A., Musci M. (2007). Retention indices in the analysis of food aroma volatile compounds in temperature-programmed gas chromatography: Database creation and evaluation of precision and robustness. J. Sep. Sci..

[22] Salas N.A., González-Aguilar G.A., Jacobo-Cuéllar J.L., Espino M., Sepúlveda D., Guerrero V., Olivas G.I. (2016). Volatile compounds in golden delicious apple fruit (Malus domestica) during cold storage. Rev. Fitotec. Mex..

[23] Rosend J., Kuldjärv R., Rosenvald S., Paalme T. (2019). The effects of apple variety, ripening stage, and yeast strain on the volatile composition of apple cider. Heliyon.

[24] Lambrechts M., Pretorius I. (2019). Yeast and its Importance to Wine Aroma—A Review. S. Afr. J. Enol. Vitic..

[25] Antón-Díaz M.J., Valles B.S., Hevia A.G., Lobo A.P. (2014). Aromatic Profile of Ciders by Chemical Quantitative, Gas Chromatography-Olfactometry, and Sensory Analysis. J. Food Sci..

[26] Wang L., Xu Y., Zhao G., Li J. (2004). Rapid Analysis of Flavor Volatiles in Apple Wine Using Headspace Solid-Phase Microextraction. J. Inst. Brew..

[27] Roberto R.M., García N.P., Hevia A.G., Valles B.S. (2005). Application of purge and trap extraction and gas chromatography for determination of minor esters in cider. J. Chromatogr. A.

[28] Pour Nikfardjam M., Maier D. (2011). Development of a headspace trap HRGC/MS method for the assessment of the relevance of certain aroma compounds on the sensorial characteristics of commercial apple juice. Food Chem..

[29] Valles B.S., Bedriñana R.P., Tascón N.F., Simón A.Q., Madrera R.R. (2007). Yeast species associated with the spontaneous fermentation of cider. Food Microbiol..

[30] Burdock G.A. (1976). Fenaroli’s handbook of flavor ingredients. Food Cosmet. Toxicol..

[31] Risticevic S., DeEll J.R., Pawliszyn J. (2012). Solid phase microextraction coupled with comprehensive two-dimensional gas chromatography–time-of-flight mass spectrometry for high-resolution metabolite profiling in apples: Implementation of structured separations for optimization of sample preparation procedure in complex samples. J. Chromatogr. A.

[32] Schwarz K.J., Boitz L.I., Methner F.-J. (2012). Enzymatic formation of styrene during wheat beer fermentation is dependent on pitching rate and cinnamic acid content. J. Inst. Brew..

[33] Bocharova O.V., Reshta S., Eshtokin V. (2016). Toluene and Benzyl Alcohol Formation in Fruit Juices Containing Benzoates. J. Food Process. Preserv..

[34] Balazs A., Tóth M., Blazics B., Héthelyi É., Szarka S., Ficsor E., Ficzek G., Lemberkovics E., Blázovics A. (2012). Investigation of dietary important components in selected red fleshed apples by GC–MS and LC–MS. Fitoterapia.

[35] Berhal C., De Clerck C., Fauconnier M.-L., Levicek C., Boullis A., Kaddes A., Jijakli H.M., Verheggen F., Sebastien M. (2017). First Characterisation of Volatile Organic Compounds Emitted by Banana Plants. Sci. Rep..

[36] Muráriková A., Ťažký A., Neugebauerová J., Planková A., Jampilek J., Mucaji P., Mikus P. (2017). Characterization of Essential Oil Composition in Different Basil Species and Pot Cultures by a GC-MS Method. Molecules.

[37] Chen Q., Song J., Bi J., Meng X., Wu X. (2018). Characterization of volatile profile from ten different varieties of Chinese jujubes by HS-SPME/GC–MS coupled with E-nose. Food Res. Int..

