# Supplementary material for: Evaluation of Volatilomic Fingerprint from Apple Fruits to Ciders: A Useful Tool to Find Putative Biomarkers for Each Apple Variety
Source: Foods. 2020 Dec 9;9(12):1830. doi: 10.3390/foods9121830 (PMC7763333; doi:10.3390/foods9121830)
Supplement: Supplementary file 1 [file foods-09-01830-s001.pdf]

foods

MDPI

Supplementary Materials

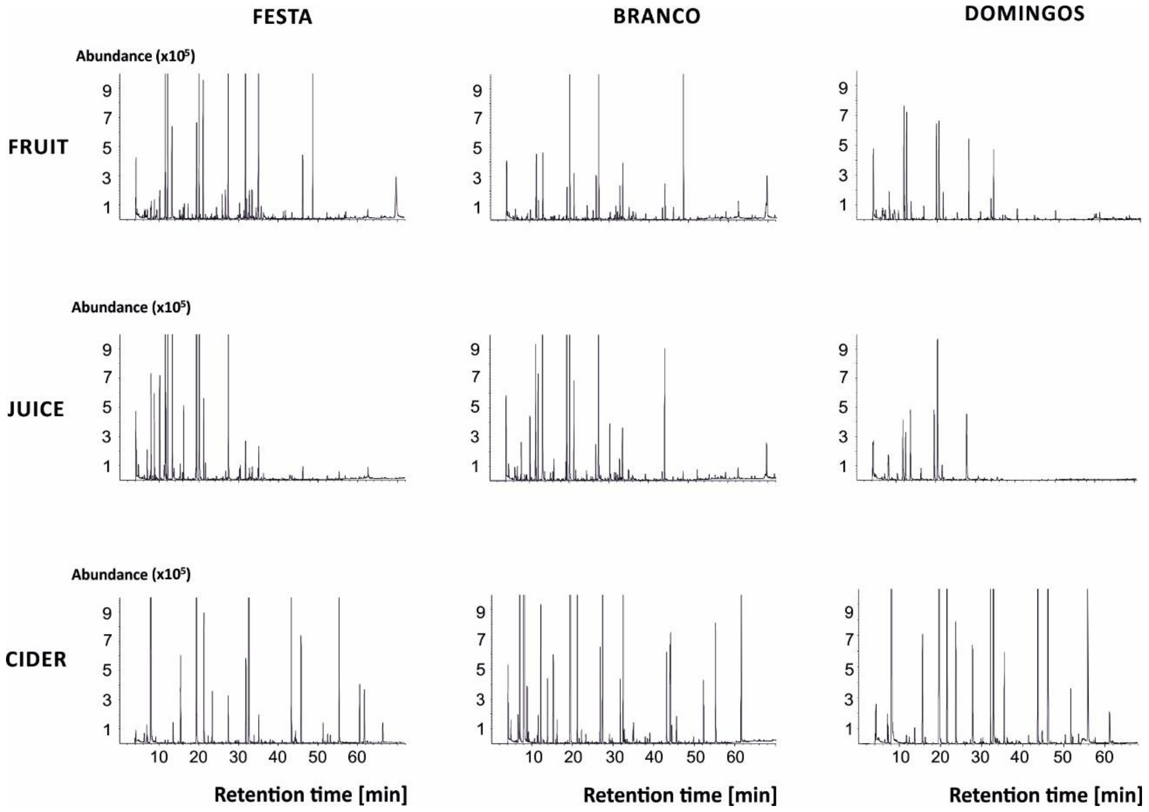

Figure 1. Total ion chromatograms obtained by HS-SPME/GC-qMS analysis of apple fruits, juices, and ciders of the different varieties (Festa, Branco, Domingos).
